# Diagnostic and Prognostic Value of miRNAs after Coronary Artery Bypass Grafting: A Review

**DOI:** 10.3390/biology10121350

**Published:** 2021-12-19

**Authors:** Ewelina Błażejowska, Tomasz Urbanowicz, Aleksandra Gąsecka, Anna Olasińska-Wiśniewska, Miłosz J. Jaguszewski, Radosław Targoński, Łukasz Szarpak, Krzysztof J. Filipiak, Bartłomiej Perek, Marek Jemielity

**Affiliations:** 1 1st Chair and Department of Cardiology, Medical University of Warsaw, 02-091 Warsaw, Poland; eb.blazejowska@gmail.com; 2 Cardiac Surgery and Transplantology Department, Poznan University of Medical Sciences, 61-701 Poznan, Poland; tomasz.urbanowicz@skpp.edu.pl (T.U.); anna.olasinska@poczta.onet.pl (A.O.-W.); bperek@ump.edu.pl (B.P.); mjemielity@poczta.onet.pl (M.J.); 3 1st Department of Cardiology, Medical University of Gdansk, 80-211 Gdansk, Poland; mjaguszewski@gumed.edu.pl (M.J.J.); rtargonski@gmail.com (R.T.); 4 Department of Clinical Sciences, Maria Sklodowska-Curie Medical Academy, 03-411 Warsaw, Poland; lukasz.szarpak@gmail.com (Ł.S.); krzysztof.filipiak@uczelniamedyczna.com.pl (K.J.F.)

**Keywords:** miRNA, coronary artery bypass grafting, coronary artery disease, diagnosis, prognosis, biomarkers

## Abstract

**Simple Summary:**

Coronary artery bypass graft (CABG) surgery is a procedure in which complex coronary arteries stenosis is treated by bypassing atherosclerotic lesions with venous or arterial grafts. CABG is associated with myocardial damage due to ischemia/reperfusion injury. In addition, postoperative complications, including perioperative myocardial infarction, atrial fibrillation and graft failure, remain important clinical challenges. So far, no reliable diagnostic and prognostic tools to predict outcomes after CABG surgery have been established. MiRNAs are noncoding, 21–24 nucleotide-long RNA particles, expressions of which alter during CABG surgery. Here, we summarize the potential clinical applicability of miRNAs as promising biomarkers to predict post-CABG procedure outcomes.

**Abstract:**

MiRNAs are noncoding, 21–24 nucleotide-long RNA particles that control over 60% of genes. MiRNAs affect gene expression through binding to the 3’-untranslated region of messenger RNA (mRNA), thus inhibiting mRNA translation or inducing mRNA degradation. MiRNAs have been associated with various cardiovascular diseases, including heart failure, hypertension, left ventricular hypertrophy, or ischemic heart disease. In addition, miRNA expression alters during coronary artery bypass grafting (CABG) surgery, which could be used to predict perioperative outcomes. CABG is an operation in which complex coronary arteries stenosis is treated by bypassing atherosclerotic lesions with venous or arterial grafts. Despite a very low perioperative mortality rate and excellent long-term survival, CABG is associated with postoperative complications, including reperfusion injury, graft failure, atrial fibrillation and perioperative myocardial infarction. So far, no reliable diagnostic and prognostic tools to predict prognosis after CABG have been developed. Changes in the perioperative miRNA expression levels could improve the diagnosis of post-CABG myocardial infarction and atrial fibrillation and could be used to stratify risk after CABG. Herein, we describe the expression changes of different subtypes of miRNAs during CABG and review the diagnostic and prognostic utility of miRNAs in patients undergoing CABG.

## 1. Introduction

Coronary artery bypass grafting (CABG) is a surgical operation in which complex coronary arteries stenosis is treated by bypassing atherosclerotic lesions with venous or arterial grafts in patients with multivessel coronary artery disease (CAD), significant coronary artery stenosis [1] and complex coronary anatomy [2]. The first CABG surgery in humans was performed in 1960 by R. Goetz [3] and, currently, around 340,000 CABG procedures per year are conducted in the United States [4]. CABG leads to a significant decrease in major adverse cardiac and cerebrovascular events (MACE) and improves health-related quality of life [5,6], especially in patients at high preoperative risk of death (ejection fraction ≤30%, presence of diabetes, unstable angina and proximal left anterior descending artery stenosis) [7]. Despite its proven benefits, the very low perioperative mortality rate (3.6%) [8] and excellent long-term survival, with the mortality rate up to 10 years after CABG only slightly higher than in the general population [9], perioperative and long-term complications are still described. Postoperative complications include infections of sternal wound, graft failure, pneumonia, atrial fibrillation, pulmonary hypertension, thromboembolic phenomena, pericardial effusion, renal injury, strokes, gastrointestinal insults and hemodynamic instability [10]. Postoperative vein graft patency is a major problem in cardiac surgery—10 years after CABG procedure, saphenous vein grafts patency reaches only from 50% to 60% and angiographic evidence of atherosclerosis occur in half of them [11,12]. Contrary to saphenous vein grafts, more than 90% of internal mammary artery grafts are still patent 10 years after CABG [13,14,15]. However, there is currently no reliable diagnostic marker, which forecasts graft patency. Current prognostic predictors, which assess clinical outcomes after CABG surgery, are based on medical and family history, lifestyle, comorbidities and performance status [16]. Thus, reliable diagnostic biomarkers that predict CABG surgery outcomes are needed. Among other biomarkers, miRNAs seem to be a promising tool to predict post-CABG outcomes.

MiRNAs are non-coding, single-stranded, 21–24 nucleotide-long RNA molecules, which are not translated into proteins [17,18]. MiRNAs play a role in suppressing gene expression via binding to 3’-untranslated region of mRNA [19] and inhibit mRNA translation or induce mRNA degradation [20]. MiRNAs are transcribed by RNA polymerase II, polyadenylated and capped with mRNA [21]. The estimated percent of genes controlled by miRNAs is over 60% [22]. The specific miRNA may have an impact on the translation of many mRNAs [23] and particular mRNA could be affected by many miRNAs [24]. Since their discovery in 1993 [25], miRNAs have been associated with the regulation of numerous biological processes underlying cardiovascular diseases, including heart failure, hypertension, left ventricular hypertrophy, or ischemic heart disease [26,27,28,29,30]. Inhibition of particular miRNA can be achieved by anti-miRs, which are modified antisense oligonucleotides [31]. Upregulation of the level of specific miRNA could be obtained through miR-mimics [32]. The changes in expression of miRNAs are triggered by inflammation [33], apoptosis [34,35] and ischemia/reperfusion injury [36], which all occur in course of CABG. In the present review, we describe the expression changes of different subtypes of miRNAs during CABG and their potential clinical applicability in patients undergoing CABG. We discuss the promising results regarding the utility of miRNA to alleviate post-CABG ischemia/reperfusion injury, inflammation and apoptosis by regulating the expression of particular miRNAs, including decreased expression of miR-195, miR-1 and miR-320 and overexpression of miR-7a/b, miR-144 and miR-133, all of which facilitate cell survival and protect from cell apoptosis. We highlight the utility of miR-499 and miR-133 to diagnose the type 5 myocardial infarction, which were shown to be more sensitive biomarkers than troponins. Moreover, we discuss miRNA expression changes in patients undergoing CABG surgery, with a special focus on patients with perioperative atrial fibrillation (AF). Especially, miR-483-5p presents significant preoperative overexpression in AF-naïve patients going on to develop post-CABG AF. Hence, it seems to be a promising biomarker, with a diagnostic accuracy of 78%. These topics are discussed to shed some lights on miRNAs’ clinical application as biomarkers and therapeutic targets in perioperative myocardial infarction, post-CABG ischemia/reperfusion injury and post-CABG atrial fibrillation.

## 2. MiRNA Utility to Diagnose Type 5 Myocardial Infarction

Perioperative myocardial infarction (PMI), which is reported in 2–8% of patients after CABG [37], increases postoperative morbidity and mortality and prolongs hospitalization. According to the Fourth Universal Definition of MI [38], PMI is classified as type 5 MI, which is defined as an elevation of cardiac troponin (cTn) values above 10 × 99th percentile of upper reference limit (URL) during the first 48 h following CABG and electrocardiographic/angiographic/imaging evidence of MI. The diagnosis of PMI could be challenging and has several limitations. Echocardiography, which is the most common and practical imaging modality, may not be diagnostic in many patients [39]. Cardiac troponins have distinguished sensitivity for the detection of myocardial damage [40] and are excellent indicators of future adverse cardiac events [41]. However, the selection of a cTn elevation of 10 × 99th as a threshold was arbitrarily chosen and, so far, there is no clear consensus on the clinically relevant level of cardiac troponin elevation. On the other hand, the elevation of cardiac troponins below this threshold is not considered in the guidelines but may still be clinically significant [39]. Finally, temporary elevation of cardiac troponins may be a common finding in a wide variety of conditions, including systemic infections [42], renal failure [43], cerebrovascular accident or atrial fibrillation [44], as well as following professional sport activity [45]. Hence, biomarkers of PMI are still needed. miR-499 and miR-133 were shown to be better biomarkers to diagnose PMI in the postoperative period (sensitivity 85.7% and specificity 93.3%, and sensitivity 89.3% and specificity 67.4%, respectively), compared to cTn (sensitivity 64.3% and specificity 86.5%) [46]. Hence, these miRNAs seem to be promising diagnostic tools of PMI.

Another problem is the time point when cTn should be measured to detect PMI. Troponins are detectable in blood after 2–4 h following myocardial injury [47], which could defer the diagnosis of PMI. On the other hand, the cTn peak is after 72 h in myocardial infarction due to a CABG procedure, which indicates that cardiac troponins should be monitored longer than 48 h postoperatively [48]. A peak high-sensitive cTnI at 6 h following CABG procedure appears to be related to the surgical process and non-specific myocardial injury, whereas a continuous increase at 24 h suggests MI [40]. MiR-499 was elevated 23-fold, 72-fold and 25-fold at 1, 3 and 6 h after declamping the aorta, respectively [46]. These results suggest that miR-499 could be considered as an early PMI biomarker, which facilitates the type 5 MI diagnosis.

## 3. Myocardial Injury during Coronary Artery Bypass Grafting

CABG performed with cardiopulmonary bypass (CPB) induces myocardial inflammation [49] and systemic inflammatory response syndrome (SIRS). Possible causes of SIRS are ischemia-reperfusion injury, operative trauma and contact of blood with the artificial surface of the extracorporeal circuit [50]. Endothelial activation and damage are crucial processes in SIRS, reflected by the presence of circulating endothelial cells (CEC) in blood–markers of endothelial activation and destruction. The number of CEC increased after CABG operation and the CPB group had a higher endothelial activation when compared to the off-pump control group [51]. Pro-inflammatory cytokine levels increased following CABG, with interleukin 6 (IL-6) and myeloperoxidase peaking at 2 h and interleukin 8 (IL-8) and tumor necrosis factor α (TNF-α) continuing to rise at 6 h. Plasma high sensitive C-reactive protein (hs-CRP) concentrations increased at 6 h and continued to rise at 48 h [40]. Increased expression of messenger RNAs IL-6 and IL-8 have associated attenuated activity and antiapoptotic effects of sphingosine kinase-1, which may contribute to myocardial apoptosis [52]. Soluble Fas molecule, which induces the apoptotic cascade, also positively correlated with the extent of inflammation and myocardial injury after CPB [53]. Serum from post-CABG patients induced apoptosis in cultured endothelial cells [54]. A higher number of apoptotic cells, assessed by caspase-3 staining, was found in biopsies taken from ventricular after aortic cross-clamp release, compared with the biopsies before on-pump CABG surgery [55]. This finding proved the hypothesis that the CABG procedure is linked with myocardial inflammation (SIRS, rise in proinflammatory cytokines) and apoptosis, which leads to myocardial injury. The review of miRNAs as markers of ischemia/reperfusion injury, inflammation and apoptosis is presented below.

## 4. MiRNAs as Markers of Ischemia/Reperfusion Injury, Inflammation and Apoptosis

Ischemia is defined as blood supply deficiency, insufficient for organ function, resulting in lack of oxygen, glucose and other substances required for metabolism. Ischemia may be symptomatically silent and may lead to life-threatening events including stroke, myocardial infarction, or peripheral vascular disease [56]. The cell damage caused by reperfusion following ischemia has led to the concept of ischemia/reperfusion injury (IRI) [57]. The expression of miRNAs is different in tissue subject to IRI and miRNAs could contribute to IRI by altering key signaling elements, which makes them promising therapeutic targets. 

### 4.1. miR-1

The level of miR-1 reduced the expression of B-cell lymphoma 2 (Bcl2) protein—a main player in the cardiomyocytes’ genetic program, favoring survival by inhibiting apoptosis in rat cardiomyocytes in ischemia/reperfusion model [58]. Moreover, inhibition of miR-1 had a positive effect on the myocardium upon IRI. Ischemic postconditioning inhibited the expression of miR-1 and down-regulation of miR-1 prevented the decrease in and redistribution of connexin 43, a protein crucial to the formation of gap junctions of cardiac cells [59]. In the IRI model, the level of connexin 43 decreased, which led to electrical conduction disturbance and arrhythmia [60]. MiR-1 overexpression may also exacerbate cardiac IRI via inhibition of protein kinase C epsilon and heat shock protein 60 [61]. Suppression of miR-1 may also contribute to the recovery of heat shock protein 90 during IRI [62]. We have previously observed that miR-1 downregulation in systolic heart failure correlated with improving symptoms [63]. Thus, downregulating the level of miR-1 may have a cardioprotective effect upon IRI. 

### 4.2. miR-7a/b

MiR-7a/b was found to be involved in IRI in myocardium and is upregulated during ischemia/reperfusion. Transfection of miR-7a/b mimic in a rat cardiac ischemia/reperfusion model decreased cardiomyocytes apoptosis and myocardium infarct size. MiR-7a/b mimic downregulates the expression of poly (ADP-ribose) polymerase (PARP), which is the major initiator of apoptosis [64]. PARP inhibition can favor cardiomyocytes’ and cardiac tissue survival and lower cardiomyocytes’ death [65]. 

### 4.3. miR-126a-5p

Another miRNA connected to ischemia/reperfusion injury is miR-126. MiR-126 expression decreases in MI and curtailment of miR-126 may protect against myocardial cell apoptosis caused by IRI [66,67]. In contrast, another study showed that overexpression of miR-126 can improve angiogenesis and cardiac function in the infarcted area of the hearts of mice [68]. In a mouse model of myocardial IRI, miR-126a-5p expression was elevated and increased the levels of lactate dehydrogenase (LDH) and creatine kinase MB (CK-MB) (damage markers) in serum. Hypoxia/reoxygenation treatment significantly increased the expression of miR-126a-5p in H9C2 cells, inhibited cell viability but increased LDH release and caspase 3 activity. Small heat shock protein (Hspb8), a protective protein in the myocardium [69], was the target of miR-126a-5p. MiR-126a-5p promoted IRI by suppressing the expression of Hspb8, which may shed light on the development of a potential therapeutic target for IRI [70]. Contrary to these results, another study showed that administration of apoptotic bodies, which were enriched in miR-126, alleviated atherosclerosis and increased the number of progenitor cells. This finding arises from the fact that miR-126 mediated the production of chemokine CXCL2, which counteracts apoptosis and recruits progenitor cells in response to DNA damage or hypoxia [71]. We have also observed, among diabetes patients, that mir-126 expression was a strong independent predictor of long-term all-cause mortality [72].

### 4.4. miR-133

MiR-133 was related to IRI in many studies. In H9C2 cells subjected to hypoxia/reperfusion injury, expression of miR-133 decreased, while transfection of miR-133 in cardiomyocytes after IRI inhibited apoptosis by targeting Death Associated Protein Kinase 2 (DAPK2) [73]. Knockdown of circulating RNA, circMAT2B, up-regulates miR-133; therefore, it reduces oxygen–glucose deprivation-induced inflammatory injury in H9C2 cells [74]. Another target of miR-133, which could be interrelated with cardiomyocyte apoptosis during IRI, was caspase-9 (CASP9)—a marker of apoptosis. CASP9 was up-regulated in the IRI group compared to the control group and was down-regulated in the ischemic postconditioning group compared to the IRI group. MiR-133 mimic decreases the level of CASP9; therefore, it may be a potential target in IRI [75]. Besides, miR-133 exerted a defense role against oxidative stress through negatively regulating caspase-3 and caspase-9 [76]. 

### 4.5. miR-144

Forkhead box O1 (FOXO1)—transcription factor which mediates cardiomyocyte apoptosis by activating the expression of inducible nitric oxide synthase—is a target for miR-144. Overexpression of miR-144 in ischemia/reperfusion rat model significantly reduced myocardial ischemic injury and apoptosis. Similar findings were observed in H9C2 cells subjected to hypoxia/reoxygenation. Transfection of miR-144 mimics reduced the mRNA and protein levels of FOXO1. These results indicate that miR-144 overexpression and, thus, FOXO1 downregulation could be a useful strategy in IRI attenuates [77]. 

### 4.6. miR-195

It was shown that miR-195 expression level is increased in myocardial IRI. Overexpression of miR-195 decreased Bcl-2 mRNA and protein expression, increased Bcl-2 associated X (Bax) mRNA and protein expression, cytochrome c protein expression and caspase-3 and caspase-9 activities. Thus, overexpression of miR-195 boosted apoptosis induced by myocardial IRI [78]. 

### 4.7. miR-320

Inhibition of miR-320 expression significantly downregulates proapoptotic particles: Bax and caspase-3 levels, upregulates anti-apoptotic Bcl-2, increases Insulin-like growth factor-1 (IGF-1) levels, which is an important regulator of cardiomyocyte homeostasis and cardiac structure, and exerts pro-survival and antiapoptotic effects [79,80]. MiR-320 inhibition target elevated IGF-1 mRNA and protein levels and countered early cardiomyocyte apoptosis of IRI [81]. Another study pointed out that the level of miR-320 and the rate of cardiomyocyte apoptosis were higher in the ischemia/reperfusion group and H9C2 cells subjected to hypoxia/reoxygenation than in the corresponding controls. Moreover, miR-320 overexpression promoted apoptosis. MiR-320 directly targets A kinase interacting protein 1 (AKIP1). The knockdown of AKIP1 inhibits apoptosis [82]. Additionally, miR-320 targets another cardioprotective particle—heat-shock protein 20 (Hsp20) [83]. Thus, miR-320 may act as a new therapeutic target for ischemic heart disease.

**Figure 1:**
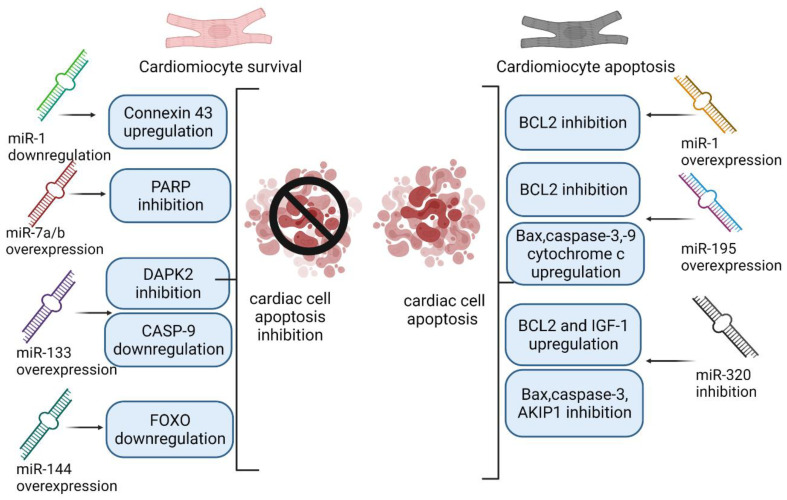
Impact of overexpression and inhibition of miRNAs on cardiac cell survival. Abbreviations: PARP (Poly(ADP-ribose)polymerase); DAPK2 (Death-associated protein kinase 2); CASP9 (Caspase 9); FOXO (Forkhead box O); BCl2 (B-cell lymphoma 2); Bax (BCL2 associated X); IGF-1 (insulin-like growth factor 1); AKIP1 (A kinase interacting protein 1).

In compliance with the results of the cited articles, regulating the expression of particular miRNAs could be a promising strategy in buffer negative effects of post-CABG ischemia/reperfusion injury. Suppressing the expression level of miR-195, miR-1 and miR-320 and overexpression of miR-7a/b, miR-144 and miR-133 may promote cardiomyocytes’ and cardiac tissue survival and protect against apoptosis. Figure 1 presents the impact of overexpression and inhibition of previously described miRNAs on cardiac cell survival. 

## 5. miRNA Expression Changes in Patients Undergoing CABG

Since the CABG procedure, especially using cardiopulmonary bypass [84], is intrinsically associated with myocardial injury, markers that indicate IRI, apoptosis and MI could be useful to monitor perioperative outcomes. MiRNA (miR-1, miR-126, miR-133, miR-199a, miR-208a and miR-499) expression changes during enumerate processes and the changes of the above-mentioned miRNAs were described in patients undergoing CABG surgery. MiRNA expression changes in patients undergoing CABG are similar. However, numerous baseline and clinical factors should be taken into account when determining the perioperative prognostic value of miRNAs, including age, gender, weight, race and body composition. 

### 5.1. miR-1

MiR-1 increased in the plasma from patients with AMI, correlated strongly with the plasma concentration of cTnI and could serve as an indicator for AMI [85]. Moreover, miR-1 presented incremental value in predicting left ventricular remodeling 6 months after acute ST-segment MI compared with clinical and cardiac magnetic resonance measurements [86] and was inversely correlated with infarct volume in patients with AMI [87]. The expression of miR-1 increased in the whole blood plasma one day after CABG surgery, as well as after CABG surgery in ACS patients [88]. The concentration of exosomal miR-1 increased at both 1 and 2 days post-CABG surgery. Exosome cargo of miR-1 was positively correlated with cTnI, but miR-1 in the whole plasma did not correlate significantly with cTnI [89].

Table 1 shows previously described miRNA expression changes in patients undergoing coronary artery grafting surgery, acute coronary syndrome patients and patients with coronary artery disease compared to control group.

### 5.2. miR-126

MiR-126 is a disease-associated miRNA richly expressed particularly in platelets and endothelial cells [102,103]. A reduction in miR-126 in endothelial cells leads to severe endothelial cell dysfunction [104], which suggests an important role of miR-126 in vasculature homeostasis. MiR-126 could serve as a potential biomarker for the complexity and severity of CAD in patients with stable angina pectoris. MiR-126 was significantly downregulated in patients with multivessel CAD and high Synergy between PCI with Taxus and Cardiac Surgery (SYNTAX) score, rather than single vessel CAD and low SYNTAX score [90]. 

The SYNTAX score is used to assess CAD severity by the points system in acute coronary syndrome (ACS), as well as in stable CAD. SYNTAX score is valuable for interventional cardiologists to select a more profitable treatment between percutaneous coronary intervention (PCI) and coronary artery bypass graft surgery [105]. Overexpression of miR-126-3p was confirmed to enhance reendothelization and reduce neointimal hyperplasia in clinically relevant vein grafts. These results indicate that miR-126-3p possesses potential clinical value for the prevention and treatment of autologous vein graft restenosis in CABG [106].

In total, 67 patients who underwent CABG surgery the levels of mir-126 and vascular endothelial growth factor-A (VEGF-A) were measured in serum, plasma and platelets at different time points [91]. VEGF-A is a proangiogenic factor, which is stored and released by activating platelets [107]. VEGF-A plays a crucial role in vascular repair and wound healing but also activates vascular smooth muscle cells (VSMC) in the intima-deficient part of vessels, which leads to graft stenosis by promoting VSMC migration and proliferation [108]. According to the study, the platelet count was decreased at day 3 after CABG, with a larger platelet change after on-pump CABG surgery compared to off-pump CABG surgery. After day 7, the platelet count increased. Thrombopoietin, which promotes platelet production [109], increased postoperatively, which could explain the surge in platelets postoperatively. Serum miR-126-3p level increased rapidly after CABG and, after day 3, decreased below preoperative levels. On day 7, the level of serum miR-126-3p was lower in patients with peripheral artery disease, who have systemic endothelial dysfunction due to atherosclerosis, than in patients without it. Platelet miR-126-3p and miR-126-5p slightly increased from day 1 to day 3, then decreased to preoperative levels. Serum miR-126-3p was significantly lower in patients with a common post-CABG complication—respiratory failure and wound complication—while platelet miR-126-3p did not differ significantly among these patients. Intra-platelet VEGF-A was significantly increased 3 days after CABG and 7 days after surgery in patients with peripheral artery disease. Serum VEGF-A level was higher 7 days after the CABG procedure. Circulating miR-126-3p levels showed a brief rise in the perioperative period. The result may be explained by the idea that the change may reflect an early compensatory mechanism by which endothelial cell function is maintained. MiR-126 modulates intracellular VEGF-A signaling by downregulating PIK3R2 and SPRED1, which suppresses the PIK3 and ERK1/2 pathways [110]. Increased serum miR-126 might inhibit VEGF-A production. Low-levels of serum miR-126p may lead to high platelet-derived VEGF-A, which is related to complications after CABG, such as graft stenosis.

Another study also showed significant reduction in miR-126 in plasma levels 4 days after CABG surgery. Moreover, miR-126 was strong associated with the level of uric acid [92]. After CABG surgery, the levels of miR-126 significantly elevated in the serum compared to pericardial fluid [111] and were higher in saphenous vein compared to aorta [112]. In contrast, in other paper, it was showed that CABG surgery did not affect the level of miR-126 [89].

### 5.3. miR-133

Cardiac expression of miR-133 was notably decreased in patients with AMI [93]. MiR-133 was shown to be involved in the process of angiogenesis, regeneration of cardiomyocytes [113] and protection of cardiac progenitor cells, which improved cardiac function in the MI model [114,115].

MiR-133a and miR-133b levels were increased after aorta declamping in CABG patients, though to a lesser extent than miR-499. Moreover, miR-133a was found to reflect the extent of myocardial injury. MiR-133 was 89.3% sensitive and 67.4% specific for the identification of PMI, compared to cTnI, which had a sensitivity of 64.3% and specificity of 86.5% for a cutoff value of 2.98 [46]. Another study also presented that, after CABG surgery was performed in 27 acute coronary syndrome patients, miR-133a level increased significantly and was associated with cTnT [88]. A strong correlation between preoperative level of EF and miR-133a levels was observed [46], while, in another study, no significant correlations between miR-133a level and preoperative left ventricle EF were detected [88]. Moreover, miR-133a is positively related to IL-6, which could suggest that miR-133a reflects the inflammatory conditions of patients during CABG surgery. Another study confirmed that miR-133 was positively correlated to IL-6 and more inflammatory markers, such as IL-8, CRP and tumor necrosis factor-alpha (TNF-α) [116]. One study assessed the potential of circulating exosomes and circulating miRNA as a novel biomarker of heart injury among patients undergoing CABG surgery [89]. Circulating exosomes, their cargo (miRNAs) and free miRNA circulating in whole plasma were correlated with the gold standard—cTns. The results of the work showed that miR-133a and miR-133b increased in the whole plasma at 24 h after CABG surgery. The concentrations of exosomal miR-133a and miR-133b increased both 24 and 48 h after CABG surgery. In this study, cardiac miRNAs in the whole blood did not correlate significantly with cTnI. CTnI was positively correlated with the plasma exosome levels and the exosomal miRNAs. This study indicates that only the plasma concentration of exosomes and their cargo of miRNA-133a/b were positively correlated with hs-cTnI. Circulating exosomes, but not plasma-circulating miRNA, could be taken into consideration as clinical biomarkers in patients undergoing CABG procedure.

### 5.4. miR-199a

Circulating miR-199a seems to be a promising biomarker in identifying PMI in cardiac surgery [46]. Lower levels of miR-199a were associated with high levels of biochemical markers related to endothelial dysfunction, inflammation and angiogenesis [117] and forecast cardiovascular events in CAD patients at long-term follow-up [94]. As mentioned above, especially on-pump CABG procedure is associated with hypoxia and oxidative stress due to ischemia/reperfusion injury. Expression of miR-199a is suppressed by hypoxia [118]. This could indicate that miR-199a should be downregulated in patients undergoing CABG surgery. MiR-199a is a cardiomyocyte-specific miRNA connected to the Sirtuin 1 (SIRT1) protein regulation. Downregulation of miR-199a contributes to the induction of SIRT1, which downregulates prolyl hydroxylase 2 (PHD2), which, in turn, stabilizes hypoxia-inducible factor 1-alpha (HIF-1-alfa), the key transcription factor of hypoxia signaling. Overexpression of HIF-1-alfa during hypoxia resulted in a smaller infarct size following ischemia/reperfusion injury [119]. Another study showed that knockdown of miR-199a during hypoxia-induced apoptosis, contrary to knockdown of miR-199a before hypoxia, surprisingly imitated pre-conditioning and saved cardiomyocytes against hypoxic injury via SIRT1 induction [120]. It has been shown that the cardioprotective effect against ischemia/reperfusion injury is provided by atorvastatin via inhibition of miR-199a-5p [121]. 

MiR-199a expression was considerably declined in patients undergoing CABG procedure in comparison to patients undergoing valve repair or replacement without CAD [95]. SIRT1 protein was greatly induced in tissues of the CABG patients compared to control. There was a negative correlation between miR-199a MACE, including all-cause death, re-hospitalization for any cardiovascular reason, heart failure symptoms ≥ NYHA II, MI, revascularization and stroke. Patients with MACE have a significantly lower level of miR-199a than uneventful patients. Between miR-199a expression and preoperative LVEF, a positive correlation was observed. This positive correlation persisted after three years of follow-up. Thus, lower levels of miR-199a are associated with worse outcomes in patients with deteriorating or even stable reduced LVEF after CABG procedure. In patients with low LVEF, a preoperatively measured miR-199a might be useful to assess individual patients’ risk for adverse events, including death. Moreover, miR-199a is a master regulator in the hypoxia-triggered pathway and downregulation of miR-199a may constitute prevention of disastrous impact of hypoxia on the myocardium.

### 5.5. miR-208a 

Cardio-enriched miR-208a is abundantly expressed during AMI [96] and presented high accuracy in discriminating patients who died suddenly due to AMI from those who succumbed without pathological cardiac involvement [97]. MiR-208b could not be detected in healthy individuals, but appeared in the plasma 1 h after myocardial injury, reaching a peak level at 3 h and then decreasing after 12 h [122]. One study measured the levels of miR-208b and miR-499-5p among 424 suspected ACS patients. The purpose of this study was to confirm the clinical value of miR-208b and miR-499-5p in myocardial infarction, heart failure detection and association with 30-days mortality. Discrimination of MI was accurate for miR-208b and miR-499-5p but lower than for troponin T. Increased miRNA levels were strongly associated with increased risk of mortality or heart failure within 30 days for miR-208b, but the association was lost when adjusting for Troponin T. The level of miR-208 was undetectable in the coronary sinus before cardioplegia but became readily detectable immediately after, which indicates that miR-208b was directly released from the myocardium due to the tissue injury [98]. Moreover, miR-208b was an independent predictor of a high SYNTAX score. In the plasma, the levels of miR-208b (and miR-499) were associated positively with the severity of CAD and could act as promising biomarkers for stratify the severity of CAD [99]. Therapeutic inhibition of miR-208a improves cardiac function and survival during heart failure [123]. Levels of miR-208 significantly increased after CABG surgery. MiR-208 was found to be associated with cardiac troponin T (cTnT). MiR-208 was also positively correlated with cardiac biomarker CK-MB, which may be explained by its cardiac specificity. IL-6 was also positively correlated with the expression of miR-208 [88]. According to these results, miR-208a could be used as a potential biomarker for the early diagnosis of cardiac injury which helps predict perioperative cardiovascular events in CABG surgery patients. 

### 5.6. miR-499

MiR-499 is muscle-enriched miRNA released from the cardiac muscle during myocardial injury into the coronary circulation [124,125,126,127]. Concentration gradients of miR-499 correlated significantly with the extent of myocardial damage as measured by hsTc [128]. The study revealed that the diagnostic accuracy of miR-499 is superior to that of cardiac TnT in elderly NSTEMI patients [100]. However, another study showed that the diagnostic power of miR-499 in distinguishing AMI reached an AUC of 0.86, quite lower than troponin AUC of 0.90 but higher than creatine kinase-MB AUC of 0.82 [124]. Another study showed that miR-499 was not superior to hsTc in early diagnosis of AMI [101].

**Figure 2:**
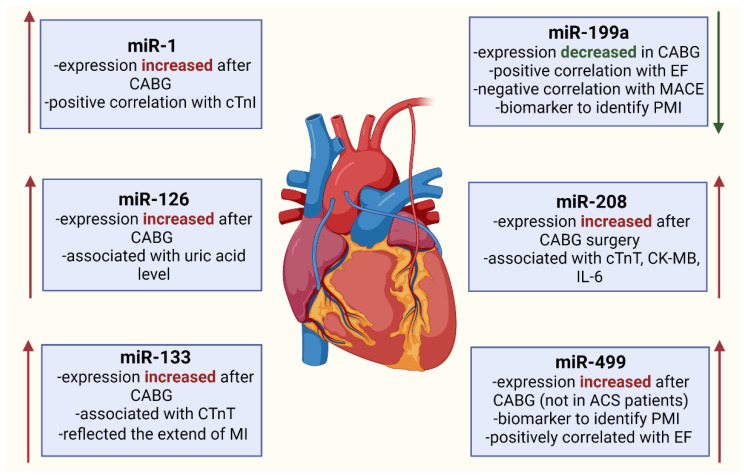
MiRNA expression changes in patients undergoing coronary artery bypass grafting (CABG). ACS (acute coronary syndrome); cTnI (cardiac troponin I); cTnT (cardiac troponin T); EF (ejection fraction); MI (myocardial infarction); PMI (perioperative myocardial infarction).

Among 29 on-pump CABG patients, the circulating levels of miR-499 were 23-fold higher at 1 h, 72-fold higher at 3 h and 25-fold higher at 6 h after aorta declamping compared to the preoperative control level. In 30% of patients, miR-499 peaked at 1 h after reperfusion and nearly 90% of patients had peak levels by 3 h, which was actually earlier than cTnI, which peaked at 6 h. The increased miR-499 level was restored back to the control value 48 h after aorta declamping. Moreover, a strong correlation between the peak values of miR-499 and cTnI was observed. Thereby, the cardiac-enriched miR-499 releasing reflected the size of myocardial injury as measured by troponin release into circulation. MiR-499 was independently associated with PMI with a sensitivity of 85.7% and specificity of 93.3%. CTnI, for a cutoff value of 2.98 ng/ml, had a sensitivity for identification of PMI of only 64.3% and a specificity of 86.5%. These results indicate that, in the early postoperative period, miR-499 is potentially a stronger biomarker than troponin in predicting PMI. In the first 48 h after CABG, the level of miR-499 was higher in the on-pump group compared to the off-pump group. The level of preoperative miR-499 significantly positively correlated with ejection fraction [46]. In addition, in the study which included only off-pump CABG patients, similar results were obtained. In 70 off-pump CABG patients, a strong positive correlation between miR-499 and plasma concentration of cTnI was observed. Moreover, the ventricle contractility (EF%) showed a significant positive correlation with miR-499 [92]. These promising outcomes are inconsistent with another study’s results, in which the level of miR-499 was not increased after CABG surgery [88]. This discrepancy may be explained by the fact that previous research works did not include ACS patients. Figure 2 shows previously described miRNA expression change in patients undergoing CABG surgery. 

## 6. MiRNA Expression Changes in Patients with Atrial Fibrillation Undergoing CABG Procedure

Postoperative atrial fibrillation (POAF) is a common cardiac arrhythmia affecting around 1 in 3 patients undergoing CABG surgery [129,130]. Expression of miRNAs (miR-483-5p, miR-208, miR-23a, miR-26a, miR-199a, miR-29 and miR-21) is altered in patients with POAF, making them interesting biomarkers.

The precise mechanism of onset and persistence of POAF has not been completely elucidated. Factors such as atrial dilation, ischemia, inflammation, atrial remodeling, trauma, autonomic imbalance and post-extracorporeal circulation could all contribute to AF after a CABG procedure [131]. At the same time, minimal invasive extracorporeal circuits have been associated with a significant reduction in the incidence of POAF [132]. POAF was associated with a lower survival rate compared to patients remaining in sinus rhythm at the 10-year follow-up (65.5% vs. 75.3%) [133]. The incidence of AF at the 10-years follow-up occurred nearly three times more often in POAF patients (15.3%) compared to healthy controls (6.4%) Moreover, POAF was associated with the development of heart failure and ischemic stroke during 9.8 years observation, which contributed to higher mortality rate in POAF patients, compared to sinus rhythm patients [134]. Currently, there are no biomarkers to predict adverse outcomes in POAF patients, which underlines the promising role of miRNA in this clinical context. Calcifications of aortic cusps and/or mitral annulus have no prognostic value for the prediction of cardiovascular events in AF patients [135].

Although POAF is such a frequent rhythm disturbance, associated with elevated mortality, its diagnosis remains problematic due to the possible asymptomatic course (silent AF) [136,137,138]. The risk factors of silent AF are age, male gender, heart failure, transient ischemic attack, diabetes, chronic kidney disease and increased NT-proB-type natiuretic peptide (NT-proBNP) concentration [139]. In 100 patients undergoing CABG surgery, silent POAF was associated with more frequent recurrences of AF than symptomatic AF during a one year follow-up [138]. Hence, it is crucial to search for novel diagnostic and therapeutic strategy in the management of silent POAF. 

According to the European Heart Rhythm Association survey, 12-lead ECG recordings at outpatient visits and 24 h Holter ECG recordings are the preferred methods to diagnose silent AF [140]. Nevertheless, the cited survey did not focus on patients with silent AF after CABG surgery. Moreover, the authors highlighted that, currently, there is no consensus on the choice of the best method for screening the patients with silent AF. Hence, other devices which facilitate the diagnosis of non-symptomatic, post-CABG AF are still needed. The long-duration ECG Holter monitoring after CABG surgery could increase the detection rate of silent AF. In the study including 100 patients after CABG, where 7-day-long Holter ECG recordings were performed, 13% of patients were diagnosed with silent AF. Only half of silent AF episodes occurred in the first four days of ECG Holter monitoring [138]. Hence, long-duration ECG Holter monitoring might enable clinicians to identify more patients with silent AF than 24 h Holter ECG recordings. The event-loop recording system is another device which offers improvements in diagnosis of silent AF. In 60 patients with acute cryptogenic stroke, the loop recorder was superior to 7-day ECG monitoring in silent AF detection (17% vs. 1.7%). However, in another study which included 740 CABG patients, 15-day postoperative event loop recorder monitoring discriminated POAF in just 10.4% of patients [141]. The upcoming study (NCT02169622), which will assess the frequency of AF after CABG surgery utilizing an implantable loop recorder, could bring new data about the usefulness of loop recorder in silent AF detection.

Targeting miRNA seems to be a promising clinical approach in preventing POAF and could reduce the hospital stays, costs, comorbidities and mortality associated with post-CABG AF. One study described pre- and post-CABG levels of three miRNAs, miR-1, miR-23a and miR-26a, in 24 POAF patients, with 24 control patients without POAF. The levels of these three miRNAs were similar among groups in the preoperative period, as were the clinical and echocardiographic profiles. The circulating levels of all three miRNAs decreased in POAF and non-POAF after CABG surgery. However, the levels of miR-23a and miR-26a were significantly lower in the POAF group during the postoperative period. The analysis of the ROC curve indicated that miR-23a and miR-26a were more efficient POAF discriminators than miR-1. The area under the ROC curve (AUC), for miR-1 was 0.57; for miR-23a, it was 0.63; and, for miR-26a, it was 0.67. The accuracy of the ROC curves was not high enough to support the use of these miRNAs as biomarkers, but this reinforces the idea that AF is triggered by a myriad of mechanisms that includes miRNA changes [142]. 

The level of another profibrotic miRNA—miR-21—which was connected to the control of the ERK–MAP kinase signaling pathway in cardiac fibroblast, was measured and could be applied in non-CABG cardiosurgery procedures [143]. Overexpression of miR-21 promotes fibroblast survival and controls the extent of interstitial fibrosis. Downregulation of miR-21 could inhibit intestinal fibrosis and protect interstitial cells against TGF-β-induced fibrogenesis and inflammation [144]. The probes from the right atrium were taken from 29 patients undergoing open-heart surgery. Patients were split into two groups, i.e., a group with preoperative AF (*n* = 16) and a group with preoperative sinus rhythm (*n* = 13). In the group described as preoperative AF (*n* = 16), patients with unsuccessful maze procedures and chronic AF (*n* = 6) were separated from the patients with successful maze operation and postoperative sinus rhythm (*n* = 10). The study results revealed that miR-21, miR-23b, miR-199b and miR-208b were significantly elevated preoperatively in the AF group, as compared with the sinus rhythm group. MiR-21 presented the most noticeable increase in the group with unsuccessful maze procedure and chronic AF (2.4-fold); the increase was smaller in the group with successful maze surgery (1.7-fold) and the smallest increase was observed in patients who had preoperative sinus rhythm (1-fold). The average area of fibrosis in patients with preoperative AF was significantly larger than that in the sinus rhythm group. The percent of fibrosis was higher in patients who undergo unsuccessful maze procedure compared to patients with successful maze surgery. Moreover, a positive relation between the fibrosis extent and expression level of miR-21 in the atrial tissue was observed. On the contrary, the plasma levels of miR-21 were decreased as compared to healthy volunteers. An inverse relationship between the level of miR-21 in atrial tissue and plasma level was detected. The expression of miR-21 in human atrial tissue is linked with atrial fibrosis and might impact AF occurrence. This indicates potential utility of miR-21, firstly, as a biomarker for cardiac surgery management and, secondly, as a potential clinical target to weaken cardiac fibrosis.

Another research group tried to find a noninvasive tool that embraced the potential of miR-29a, an miRNA-modulating fibrosis [145], to identify patients at high risk for new-onset POAF. They measured concentrations of biomarkers associated with activation of fibroblast and collagen turnover, extracellular matrix and miRNA-29 in serum from preoperative blood samples. The collagen cumulation has been documented in patients with AF as compared with patients with sinus rhythm [146]. Moreover, atrial fibrosis is a frequent structural change in atrial tissue, that affects the occurrence of AF [147]. Biomarkers reflecting collagen synthesis and degradation, such as carboxyl-terminal pro-collagen I (PICP), amino-terminal procollagen III (PIIINP) and carboxyl-terminal telopeptide of collagen I (CITP), were proposed to identify patients at high risk of POAF [148], but they are not sufficient as a single marker [149]. In this study, 90 patients without prior AF were examined. Thirty-four patients who developed AF were older than non-POAF patients, but with no significant differences in baseline comorbidities, left atrium size, or ventricular function. The level of PIIINP, the marker of collagen III synthesis, was elevated in POAF patients compared to non-POAF patients. These PIIINP expression changes correlated with a reduction in miR-29 levels and with atrial fibrosis extent. PICP, the marker of collagen I synthesis, increased in POAF patients but did not reach statistical significance. CITP did not differ between POAF and non-POAF groups. The author concluded that, in AF-naïve patients, combining age, the biomarker of collagen III synthesis (PIIINP) and miR-29a provided a model that identified POAF patients with higher predictive accuracy [150]. Another study describes the levels of miR-199 and miR-195 in 63 patients referred for isolated CABG surgery with cardiopulmonary bypass. MiR-199a was previously defined as miRNA deregulated in patients who develop paroxysmal AF after cardiac operation [151]. Both miR-195 and miR-199a regulate the expression of the SIRT1 protein, the enhanced expression of which is associated with the occurrence of AF [152]. The authors investigated whether SIRT1 regulating miR-195 and miR-199a are involved in the pathogenesis of AF. The levels of miR-199a and miR-195 were measured in tissue probes taken from the right atrial appendage (RAA) before starting the cardiopulmonary circulation. After CABG surgery, all patients were rhythm-monitored until discharge and randomized to a POAF (*n* = 20) or a sinus rhythm (*n* = 43) group. The expression of miR-199a in the POAF group was significantly decreased compared to sinus rhythm patients. MiR-195 was also less expressed in the samples of the POAF group, but without showing a significant difference compared to the non-POAF patients. SIRT1 was significantly induced in patients with POAF. The outcomes of this study revealed that altered expression of SIRT1 protein is related to the occurrence of POAF and could be useful as a biomarker for cardiac surgery management. Moreover, the decreased steady state of miR-199a, with subsequent activation of the SIRT1 protein, might predict postoperative atrial fibrillation [153].

**Figure 3:**
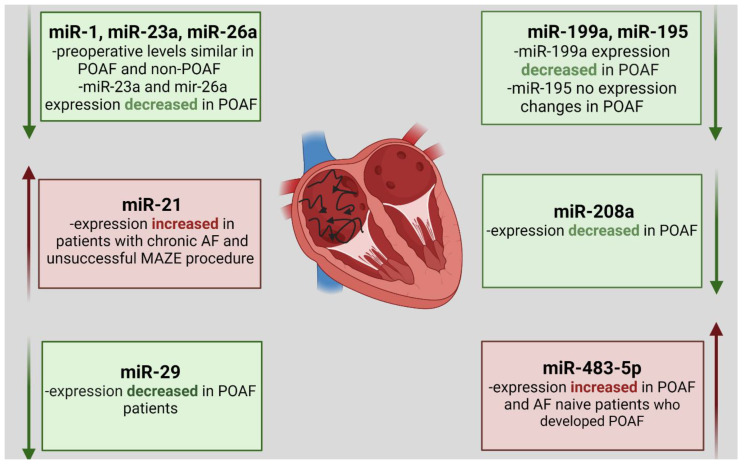
MiRNA expression changes in patients with postoperative atrial fibrillation (POAF) after coronary artery bypass grafting (CABG).

The levels of miR-483-5p and miR-208a were compared in 13 patients who developed POAF and 21 patients who maintained sinus rhythm after CABG procedure. MiR-208a was the most underexpressed and miR-483-5p was the most overexpressed in the atrial myocardium of POAF patients compared with those who maintaining sinus rhythm. MiR-483-5p presented significant overexpression in the preoperative serum of AF-naive patients, going on to develop AF in the postoperative period. Circulating miR-483-5p seems to be a promising biomarker for POAF discrimination, with a diagnostic accuracy of 78%. This diagnostic accuracy is far higher than pre-operative pro-BNP, C-reactive protein and peripheral WBC count which have been reported to have diagnostic accuracies of 61%, 68% and 69%, respectively [154]. Figure 3 presents previously described miRNA expression changes in patients with atrial fibrillation after CABG.

## 7. Perioperative Prognostic Value of miRNAs

Circulating miRNAs could serve as diagnostic and prognostic biomarkers for cardiovascular events in CABG patients. As underlined before, recognition of type 5 MI corresponding to the Fourth Universal Definition of MI remains challenging. Additional markers, which allow all PMI to be recognized early, with high sensitivity and specificity, appear to be necessary. MiRNAs seem to be a promising tool in the establishment of post-CABG myocardial infarction diagnosis, especially miR-133 and miR-499, as they can distinctly forecast the occurrence of PMI, with sensitivity and specificity higher than cardiac TnI [46]. MiR-208 could also be used as a biomarker for the early diagnosis of cardiac damage in CABG surgery patients. Contrary to the levels of miR-133 and miR-499, the level of miR-208 positively correlated with cardiac biomarker CK-MB and may be more relevant to cardiac failure, because muscle injures influence the expression of the other two miRNAs [88]. MiR-208 and miR-499 appear to be promising as factors of myocardial damage after cardiac surgery not only in adults but also in children [155].

Affecting the levels of various miRNAs could be a useful strategy in PMI prevention. As mentioned above, downregulation of miR-195, miR-1 and miR-320 and overexpression of miR-7a/b, miR-144 and miR-133 could attenuate IRI, thus preventing cardiac muscle from cell apoptosis, favoring cell survival and counteracting PMI. In addition, knockdown of miR-199a before hypoxia imitated pre-conditioning and protected cardiomyocytes against hypoxic damage [120]. 

MiR-126 could play a role in CABG grafts patency. Through upregulation of miR-126, the production of platelet-derived VEGF-A decreased, which provided protection from by-pass closure [91]. Platelet-derived MiR-223, in turn, could serve as a predictor for bleeding during and after off-pump CABG surgery [156].

MiRNAs have shown to be correlated to EF and NYHA status in perioperative studies. In particular, the preoperative miR-199 level could be helpful in patients with low LVEF to stratify individual patients’ risk for adverse events [95]. MiR-133 expression in the right atrial appendage was decreased by 20% in NYHA functional classes III and IV compared to classes I and II and decreased in patients with NT-proBNP concentrations exceeding 1800 pg/mL. The decreased miR-133 expression indicated heart failure in patients undergoing CABG procedure [157]. 

The ability to recognize patients who are prone to develop post-CABG atrial fibrillation is crucial in POAF prevention and in reducing mortality and utilization of health care resources. Targeting miRNAs could be a promising solution in the management of postoperative atrial fibrillation. MiR-483-5p and miR-199a could be used to distinguish patients in danger of POAF before it occurs [153,154]. Combining age, PIIINP and miR-29a provided a model which identified patients with high predictive accuracy [150]. MiR-23a and miR-26a may be involved in the underlying biology of POAF development and the regulation of the levels of this miRNA could inhibit vulnerability to post-CABG AF [142]. 

In pediatric heart surgery, miR-1 could precipitate the recognition of patients with cardiopulmonary bypass-related ischemic complications, who required more strenuous support. At the end of the operation, the level of miR-1 positively correlated with early severe cardiovascular events, intensive care stay, ventilation index and high vasoactive-inotrope score [158]. 

## 8. Economic Aspects of miRNA-Based Diagnostic and Prognostic Strategies

The economic aspect regarding the use miRNAs as biomarkers or therapeutic targets in cardiovascular diseases is poorly investigated in the literature. However, the cost-effectiveness of miRNAs as a biomarker was assessed in non-cardiovascular diseases. The use of miRNA blood tests for gastric cancer screening not only improved the early diagnostics, but also was cost-effective, for the price of USD 70,000/quality-adjusted life-year in more than 95% of iterations [159], and cost-saving, with USD 200/patient [160]. It is essential to select low-cost, sensitive and fast-throughput methods to detect miRNAs. One study described a method called iLluminate-miRNA, which improved assay time from 7 h to 1 h, improved the sensitivity of the miRNA method detection and reduced costs from USD 2000 to USD 60 [161]. This promising results indicate that applying miRNAs in the prognosis of CABG outcomes could be a cost-effective strategy, but it needs to be confirmed by future research.

## 9. Conclusions

MiRNAs are emerging potential targets for therapeutic intervention as well as novel clinical biomarkers during CABG surgery, but the results regarding their clinical application require confirmation in future studies. Impacting targeted mRNAs expression and protein synthesis by up- and down-regulating miRNA seems to be an effective strategy in decreasing ischemia/reperfusion injury, preventing POAF and extending the recognition of patients who are at risk or have suffered from PMI. Our manuscript summarizes the hitherto available studies regarding the diagnostic and prognostic value of miRNAs after CABG surgery. The studies related to our topic are already available; however, we underline the correlation between CABG surgery and IRI and highlight the problem of POAF in patients undergoing CABG surgery. This is, to the best of our knowledge, the first review which organizes all these data. We describe the changes in the level of miRNAs (miR-1, miR-133a, mir-208 and miR-499) that are predominantly expressed in cardiac tissue. However, the studies discussed in our manuscript include small patients’ numbers, various sampling time frames, distinct methods for miRNA quantification and co-morbidities. Moreover, the perioperative prognostic value of miRNAs could be affected by different physiologically variables, including sex, age, weight, race and body composition. Therefore, additional larger studies in both animal models and human clinical trials are needed to validate miRNAs as clinical biomarkers for diagnosis and targets for treatment. Moreover, there is a need to assess the cost-effectiveness of miRNAs as biomarkers and therapeutic targets after CABG.

## Figures and Tables

**Table 1:** miRNA expression changes in patients undergoing coronary artery grafting surgery, acute coronary syndrome patients and patients with coronary artery disease compared to control group.

miRNA	Patient Group	Control Group	Outcome	Ref.
mir-1	17 AMI patients	25 healthy adults	miR-1 expression increased in AMI patients and correlated with cTnI.	[85]
	80 STEMI patients: 22 with adverse LV remodeling, 58 without adverse LV remodeling	n/a	miR-1 predicts LV remodeling in patients with STEMI alone and shows incremental prediction value compared to clinical and magnetic resonance imaging.	[86]
	44 AMI patients	18 healthy adults	miR-1 increased in AMI patients and was inversely correlated with infarct volume (CMR marker of adverse ventricular remodeling).	[87]
	29 CABG patients with ACS	n/a	miR-1 increased after CABG surgery, did not correlate with cTnT.	[88]
	21 CABG patients	n/a	Plasma and exosome miR-1 increased after a CABG procedure. Exosomal but no plasma-circulating miR-1 positively correlated with cTnI.	[89]
miR-126	140 CAD patients	40 patients without CAD	The miR-126 level was lower in patients with multivessel CAD and higher SYNTAX score but not dramatically downregulated in patients with one vessel CAD and low SYNTAX score compared with the control.	[90]
	67 CABG patients	n/a	miR-126-3p level in serum increased rapidly after CABG and then decreased below preoperative levels. Seven days after CABG surgery, miR-126-3p level was higher in patients with peripheral artery disease (PAD), compared with patients without PAD.	[91]
	70 off-pump CABG patients	n/a	miR-126 was downregulated 4 days after CABG surgery and correlated strongly with the level of uric acid.	[92]
miR-133	50 patients with MI	8 healthy adults and 9 fetuses	miR-133a was downregulated in patients with MI compared to control.	[93]
	30 on-pump CABG patients	n/a	miR-133b level increased after declamping in CABG patients. Moreover, miR-133a was found to reflect the extent of myocardial injury. miR-133 was 89.3% sensitive and 67.4% specific for the identification of PMI compared to cTnI, which had a sensitivity of 64.3% and specificity of 86.5% for a cutoff value of 2.98.	[46]
	27 ACS patients undergoing CABG surgery	n/a	After CABG surgery, miR-133a level increased significantly and was associated with cTnT.	[88]
	21 CABG patients	n/a	Plasma and exosomal miR-133a and miR-133b increased after CABG surgery. Exosomal but not plasma-circulating miR-133a and mir-133b correlated positively with cTnI.	[89]
miR-199	181 patients with stable CAD	n/a	Increased expression of microvesicles containing miR-199a but not freely circulating miR-199a was significantly associated with a lower major adverse cardiovascular event rate.	[94]
	68 CABG surgery patients	34 patients undergoing heart valve surgery	The level of miR-199a in CABG patients was significantly reduced compared to the control group. Patients with a major adverse cardiac event had a significantly lower level of miR-199a than uneventful patients.	[95]
miR-208	62 MI patients	18 cases of traumatic death without cardiac pathology	miR-208 was highly expressed in AMI patients.	[96]
	19 cases of death due to AMI 25 cases of sudden cardiac death	n/a	miR-208 presented high accuracy in discriminating patients who died suddenly due to AMI from those who succumbed without pathological cardiac involvement.	[97]
	424 patients with suspected ASC	n/a	miR-208 was higher in MI patients and correlated with LVEF. The increased miR-208 level was strongly associated with an increased risk of mortality or heart failure within 30 days.	[98]
	195 CAD patients	n/a	High plasma levels of miR-208 were positively associated with the severity of CAD and plasma miR-208b could act as a potential biomarker for estimating the severity of CAD.	[99]
	27 ACS patients undergoing CABG surgery	n/a	miR-208 significantly increased after CABG surgery and was found to be associated with cTnT, CK-MB and IL-6.	[88]
miR-499	92 NSTEMI patients, 81 patients with CHF without AMI	99 healthy patients	The diagnostic accuracy of miR-499 was superior to that of cardiac TnT in elderly, NSTEMI patients.	[100]
	41 AMI patients, 32 SAP patients	10 healthy patients	Serum miR-499 expression level at different time points was significantly higher in the AMI group than in the SAP group and control group. miR-499 was not superior to hs-cTnI as myocardial marker in the diagnosis of early AMI.	[101]
	30 on-pump CABG patients, 30 off-pump CABG patients and a prospective cohort of 120 on-pump CABG patients	n/a	miR-499 had higher sensitivity and specificity than cTnI for identifying PMI in cardiac surgery and is a novel, early biomarker for identifying perioperative myocardial infarction in cardiac surgery.	[46]
	70 off-pump CABG patients	n/a	A strong positive correlation between miR-499 and plasma concentration of cTnI and miR-499 and ventricle contractility (EF%) was observed.	[92]
	29 ACS patients undergoing CABG surgery	n/a	The level of miR-499 was not increased after CABG surgery in ACS patients.	[88]

Abbreviations: MI (myocardial infarction); AMI (acute myocardial infarction); ASC (acute coronary syndrome); STEMI (ST elevation myocardial infarction); NSTEMI (non-ST-elevation myocardial infarction); CAD (coronary artery disease); LV (left ventricle); LVEF (left ventricle ejection fraction); cTnI (cardiac troponin I); cTnT (cardiac troponin T); hs-cTnI (high sensitivity cardiac troponin I); CABG (coronary artery grafting); CHF (congestive heart failure); SAP (stable angina pectoris); CK-MB (creatine kinase-MB); CMR (cardiovascular magnetic resonance imaging); IL-6 (interleukine 6).

## Data Availability

Not applicable.
